# Still Dazed and Confused: Demographic Drift and the Persistence of Sampling Bias in Cannabis Research

**DOI:** 10.3390/life16101712

**Published:** 2026-10-13

**Authors:** Thorsten Rudroff

**Affiliations:** Turku PET Centre, University of Turku and Turku University Hospital, FI-2052 Turku, Finland; thrudr@utu.fi

**Keywords:** cannabis research, sampling bias, dual diversity framework, aging/older adults, neuroimaging heterogeneity

## Abstract

Cannabis use has shifted dramatically over the past two decades, with the fastest growth occurring among adults aged 65 and older. The clinical trial, neuroimaging, inflammation, and psychiatric outcome literatures on cannabis have not kept pace with this change. This narrative review argues that cannabis research suffers from both dimensions of a Dual Diversity problem: a population diversity deficit, in which study samples remain skewed toward young, healthy, often university-recruited participants relative to actual users, and heterogeneity suppression, in which a demographically varied user population is analyzed as a single, uniform exposure group. This mismatch is traced across five domains. A documented clinical trial diversity gap has been quantified but not yet addressed. Patients using cannabis for neurological disease and chronic pain self-medicate ahead of an evidence base built for a different population. Neuroimaging studies of older-adult cohorts report divergent structural findings that may reflect participant and exposure heterogeneity, among other possible explanations. Inflammatory and endocannabinoid physiology change with age in ways young-skewed samples rarely capture. Psychosis-risk research remains concentrated in adolescence despite implications for older, more recent initiates. The review closes with concrete recommendations, including population-benchmarked reporting, age-stratified enrollment modeled on ICH E7, heterogeneity-preserving analysis, and a minimum exposure descriptor set.

## 1. Introduction

Much of the foundational cannabis neuroimaging and clinical trial literature was designed when the typical cannabis user was young, often male, often recruited from a university campus, and rarely taking more than one other medication. That user still exists, but he is no longer representative. Past-month cannabis use among US adults aged 65 and older rose from 4.6 percent to 7.0 percent between 2021 and 2023 alone, a two-year increase that outpaces growth in nearly every other age group [1]. Among veterans aged 65 to 84, one in ten now reports past-30-day cannabis use, and more than a third of those users meet criteria for cannabis use disorder [2]. The population using cannabis has changed faster than the population being studied to understand it.

This is not simply a story about legalization expanding access, though it is partly that. It is a story about a research base that has not renegotiated its sampling assumptions to match the population it claims to describe. Barkholtz and Bates demonstrated this directly in 2023, quantifying a measurable gap between the demographics of participants enrolled in cannabis clinical trials and the demographics of people who report using cannabis in national survey data [3]. That finding, three years old at the time of this writing, has not yet been extended to neuroimaging, inflammation, or psychiatric outcome research, nor connected to a general account of why the gap persists and what it costs the field.

I have argued elsewhere that demographic composition should be treated as a validity threat, not folded into the general category of confounding, because a validity threat implies that the finding may not generalize at all rather than merely needing statistical adjustment [4]. In formal epidemiological terms, enrolling a sample that does not represent the target population introduces selection bias, which limits external validity and transportability. When the response to an exposure differs across age groups, that difference is effect modification. Neither is a confounding problem that statistical adjustment alone can resolve. Cannabis research is a useful test case for that argument, because the gap between who uses cannabis and who is studied using cannabis is unusually large, unusually well documented, and unusually fast moving. The title of this piece is not nostalgic. Cannabis research is not confused in the way it was during the prohibition era of stigma and scientific neglect. It is careful, methodical work whose samples increasingly differ from the population of people who use cannabis, at a moment when that population has become older, more medically complex, and harder to ignore.

What follows examines that mismatch across five domains—the documented diversity gap itself, who uses cannabis for neurological disease and chronic pain, neuroimaging, inflammation and immune function, and psychiatric outcome research—before closing with concrete recommendations for what a research base that had kept pace would look like. This is a narrative review, and Section 2 describes how the literature was identified.

## 2. Methods

This is a narrative review. It was not preregistered, it is not intended to be exhaustive, and it does not support pooled estimates. Reporting follows the SANRA checklist for narrative reviews [5].

Sources were identified through targeted searches of PubMed from 1 January 2020 to 6 October 2026, combining concept blocks for cannabis or cannabinoids, older adults or aging, sampling or trial demographics, and each domain reviewed (clinical trial diversity, neurological disease and chronic pain, neuroimaging, inflammation and immune function, psychosis risk, and polypharmacy and screening). Full search strings are in the Supplementary Materials. Eligible sources were peer-reviewed human studies, systematic reviews, and regulatory documents in English that reported participant age composition or age-related outcomes. Preclinical-only studies, case reports, and studies without age information were excluded. Preprints were included only when labeled as such and were not used to support a central claim.

Because the quantitative anchor of the review is Barkholtz and Bates [3], additional sources were found by forward and backward citation searching from that paper and from the primary studies in each domain. Older foundational sources, such as ICH E7 and NSDUH trend data from 2002, were identified through citation searching and regulatory sources and fall outside the dated search window. I also searched specifically for findings that contradict the argument, such as studies reporting no age differences or favorable outcomes in older users. The combined cannabis and age search returned 763 unique records. Titles were screened with the assistance of an AI model, followed by author review. Of the 763 records, 102 were excluded (72 preclinical or non-human studies, 23 adolescent- or young-adult-only studies, and 7 case reports or series), 456 were carried forward as candidates, and 205 had no cannabis term in the title and were not systematically assessed. Screening was at the title level only, and abstracts were not systematically screened. Of the 26 journal articles cited in this review other than reporting guidelines, 14 were retrieved by these searches, and the remainder were found by citation searching or from the author’s knowledge of the field. Selection and appraisal were conducted by a single author without formal risk-of-bias assessment, which is acknowledged in Section 12.

## 3. Who Uses Cannabis Now

The scale of the demographic shift is easy to understate by citing a single percentage, so it is worth sitting with the trajectory rather than a snapshot. Using National Survey on Drug Use and Health data spanning 2002 to 2021, Barkholtz and Bates documented a rise in past-month cannabis use among adults 65 and older of roughly 2066 percent, and a rise of roughly 472 percent among adults 50 to 64, over that same nineteen-year window [3]. No other age band comes close. By 2021, past-month cannabis users were 56.6 percent male and 43.4 percent female, a narrower sex gap than the stereotype of the young male user would predict, and the age distribution had become genuinely broad: 24.4 percent were 26 to 34 years old, 24.1 percent were 35 to 49, 22.4 percent were 18 to 25, and 17.6 percent were 50 to 64 [3]. Cannabis use is no longer concentrated in early adulthood. It is spread across the adult lifespan, with the fastest growth happening at the older end of it.

This is the empirical anchor for everything that follows, and it plays the same structural role in this piece that the finding of biological sex explaining roughly 30 percent of globus pallidus metabolic variance, against a symptom category explaining effectively none, has played in my prior work on fatigue neuroimaging. In both cases, a single well-replicated demographic finding exposes a gap between the variable the field has organized its sampling around and the variable that predicts the outcome. In fatigue neuroimaging, the field has sampled by diagnostic category and underweighted sex. In cannabis research, the field has sampled by convenience, largely young age and largely good health, and underweighted age.

I have proposed elsewhere that this kind of mismatch is best understood through a Dual Diversity framework, which identifies two compounding and independent threats to validity in biomedical research [4,6]. The first is population diversity deficit, or WYHU sampling: research samples drawn disproportionately from participants who are young, healthy, and university-recruited, relative to the population the findings are meant to describe. In epidemiological terms, this is selection bias affecting external validity and transportability. The second is neurobiological or clinical heterogeneity suppression: the practice of treating a diagnostic or exposure category as internally uniform, so that meaningful subgroup differences, by age, sex, comorbidity, or severity, are averaged away rather than reported. In epidemiological terms, this is the failure to estimate or report effect modification. In this review, heterogeneity suppression applies both to participants (age, sex, comorbidity, polypharmacy) and to the exposure itself (Section 4). Neither threat is fully captured by the standard statistical vocabulary of confounding, because a confounder is something a good model can adjust for. A validity threat of this kind means the finding may not describe the population it claims to describe at all, no matter how the model is specified [4].

Cannabis research is an unusually clean case study for this framework because it exhibits both failure modes at once, and because the mismatch is worsening in real time rather than sitting static. WYHU sampling shows up directly in clinical trial enrollment, which continues to skew younger and healthier than the survey-documented user population, as the diversity gap analysis in Section 5 makes explicit. Heterogeneity suppression shows up in how the literature treats cannabis user as a single exposure category across studies that pool 22-year-old recreational users with 74-year-old patients managing chronic pain on four other medications, or that compare a UK Biobank cohort against a US clinical cohort without treating the underlying differences in product potency, legal market structure, and comorbidity profile as data rather than noise. I return to this second example directly in the neuroimaging section, where two well-powered studies of older cohorts with overlapping but not identical age ranges report divergent findings, a divergence that may reflect heterogeneity suppression among other possible explanations (Section 7).

The remainder of this piece works through five domains in which this dual mismatch appears: the clinical trial diversity gap itself, documented but not yet acted upon; who actually uses cannabis to manage neurological disease and chronic pain, and how poorly that population is captured by trial-based evidence; neuroimaging, where the age-related contradiction is sharpest; inflammation and immune function, where the endocannabinoid system’s age-dependent behavior is only beginning to be taken seriously; and psychiatric outcome research, where an entire subfield remains concentrated in adolescence and young adulthood even as its own THC-potency argument implies older users are facing a materially different drug than the one their generation grew up avoiding or using. A field that had kept pace with its own population would look different in each of these five places. Section 11 describes what that would look like.

## 4. Target Populations and Exposure

Representativeness is a property of a sample relative to a specific question, not an absolute quality. In epidemiological terms, a sample that differs from the target population on a variable that modifies the effect is a selection problem that limits external validity and transportability, and pooling strata that respond differently conceals effect modification. Confounding, by contrast, is an internal validity problem that adjustment can address. The two dimensions of the Dual Diversity framework [4,6] correspond to these concepts: population diversity deficit is selection bias affecting transportability, and heterogeneity suppression is the failure to estimate or report effect modification. The relevant benchmark depends on the question being asked, and Table 1 pairs each research question used in this review with its target population and an appropriate benchmark. National survey data are appropriate for questions about who is exposed and whether dosing and safety findings transfer across ages, whereas an efficacy question for a specific condition should be benchmarked against people with that condition who use cannabis.

**Table 1:** Target population and benchmark depend on the research question.

Research Question	Target Population	Appropriate Benchmark	Example in This Review
Who is exposed in the community?	Adults who use cannabis	National surveillance (NSDUH)	Section 3
Is dosing and safety transferable across ages?	Adults of all ages who use cannabis, including older adults	Age distribution of users in surveillance data	PK/PD trials, Figure 1
Does cannabis relieve symptoms of a condition?	Patients with that condition who use or would use cannabis	Condition-specific registries and surveys	MS and Parkinson disease cohorts, Section 6
How is use related to brain structure in aging?	Community-dwelling older adults with a defined exposure history	Population cohort age profile, stratified by exposure	UK Biobank and Colorado, Section 7
Does risk vary with age at initiation?	People starting use, by age at initiation	Incidence cohorts by age of onset	Adolescent cohorts versus late-life initiators, Section 9
What interaction risk exists at the point of care?	Older adults using cannabis with prescribed medicines	Pharmacy and EHR medication data	Section 10

**Figure 1:**
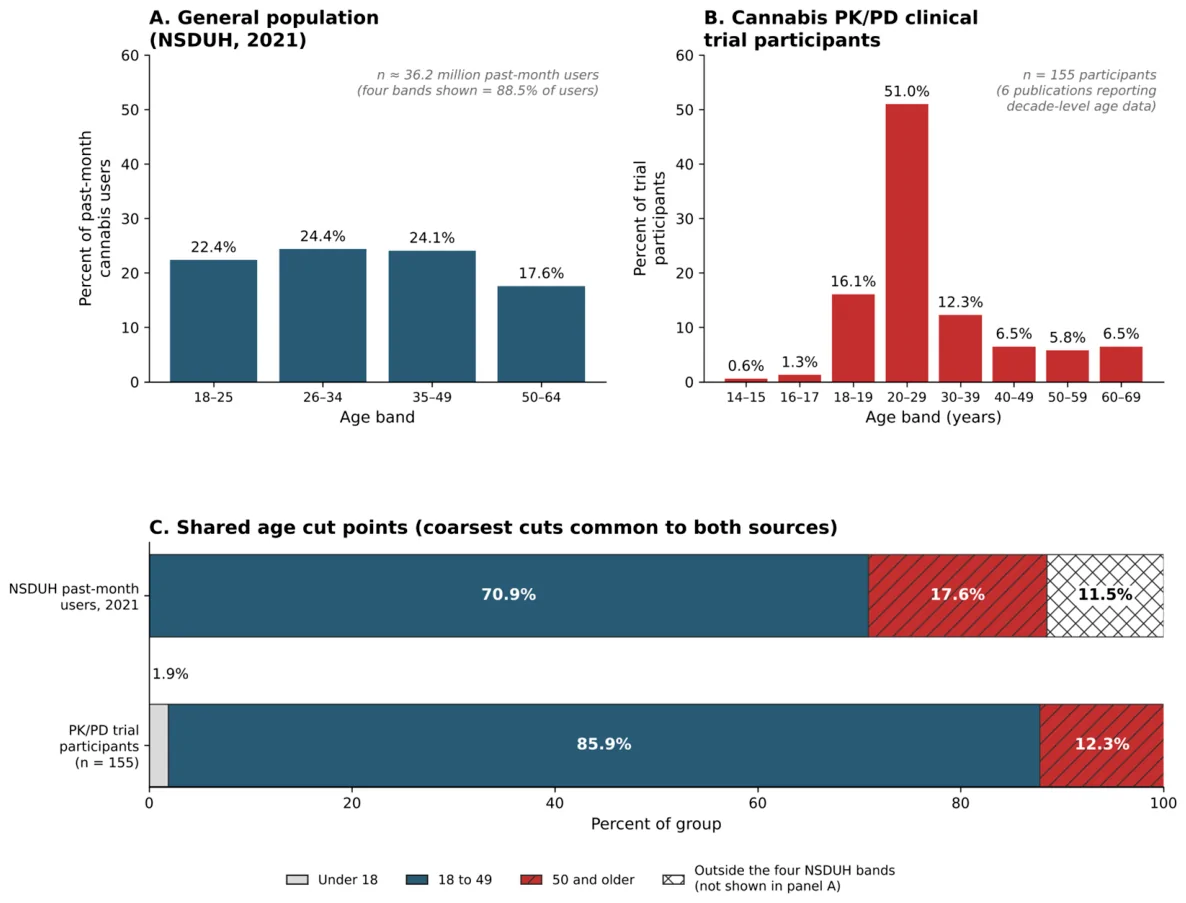
Age distribution of cannabis users in the general population versus cannabis pharmacokinetic and pharmacodynamic clinical trial participants [3].

(A) Age distribution of US adults reporting past-month cannabis use in 2021 (approximately 36.2 million people), drawn from National Survey on Drug Use and Health data as reported by Barkholtz and Bates [3]. Use is broadly distributed across four adult age bands: 18 to 25 (22.4 percent), 26 to 34 (24.4 percent), 35 to 49 (24.1 percent), and 50 to 64 (17.6 percent). These four bands account for 88.5 percent of past-month users; the remaining 11.5 percent fall in age groups not shown.

(B) Age distribution, by decade, of participants enrolled in cannabis pharmacokinetic and pharmacodynamic clinical trials with prior cannabis exposure, drawn from the subset of 155 participants across six publications reporting age at decade-level granularity, as identified in the same source [3]. Just over half of these participants (51.0 percent) fall within the 20 to 29 age band, with markedly smaller representation in every decade beyond it. The bands include participants aged 14 to 17 (1.9 percent combined).

(C) Comparison on the coarsest age cut points shared by both sources. NSDUH past-month users [3]: 18 to 49 years, 70.9 percent; 50 to 64 years, 17.6 percent; outside the four reported bands, 11.5 percent. PK/PD trial participants: under 18 years, 1.9 percent; 18 to 49 years, 85.9 percent; 50 to 69 years, 12.3 percent (no participants aged 70 or older were reported). Percentages may not sum to 100 because of rounding.

Age bands differ between panels because each panel reflects how its underlying source reported age data (four-category NSDUH age bands in panel A; decade-level bands as extracted from the clinical trial literature in panel B); the two panels are not intended to share a single axis scale. All bars are individually labeled with their percentage value and are distinguishable by position and label alone, without reliance on color, for accurate interpretation in black-and-white print. Panel C uses the coarsest cut points that both sources share. Panel B rests on a small subsample (n = 155, six publications) and illustrates enrollment in PK/PD trials only. Data source for both panels: Barkholtz H, Bates M. “Measuring the diversity gap of cannabis clinical trial participants compared to people who report using cannabis” [3].

Heterogeneity suppression has two layers: participant heterogeneity (age, sex, comorbidity, polypharmacy) and exposure heterogeneity. Cannabis use is not a single exposure, and Table 2 lists the dimensions along which exposure varies, why each matters, and the minimum descriptor a study should report.

## 5. The Diversity Gap Is Already Documented, Just Not Named

The gap described in the previous section is not a matter of inference. Barkholtz and Bates measured it directly. Using National Survey on Drug Use and Health data on past-month cannabis use across population subgroups from 2002 to 2021, they extracted participant demographics from peer-reviewed cannabis clinical trials and compared the two distributions directly, and adjusted for age, sex, and race and ethnicity, against the population of people who actually report using cannabis [3]. The comparison is the useful part. Most discussions of underrepresentation in cannabis research gesture at the problem qualitatively (trials probably skew young, trials probably skew white), without a population-level benchmark to measure the skew against. Barkholtz and Bates supplied that benchmark, and it functions as the empirical backbone of the diversity gap claim this piece is built around.

What their analysis did not do is equally important to state plainly. It did not extend into neuroimaging, where sample composition determines what a scanner is measuring rather than merely to whom a treatment effect applies. It did not extend into inflammation or immune-function research, where age-related immunosenescence makes demographic composition mechanistically relevant rather than a matter of external validity alone. It did not extend into psychiatric outcome research, where the field’s own risk factors, including age of onset, product potency, and frequency, are themselves demographic variables that a pooled cannabis user category obscures. Moreover, because the analysis relies on data only through 2021, it precedes the recent sharp acceleration in older-adult use documented earlier. The gap that Barkholtz and Bates measured has plausibly grown wider rather than narrower since their data window closed, because clinical trial recruitment practices evolve far more slowly than the demographics of who is willing to use a drug now legal in most states. The NSDUH benchmark answers questions about who is exposed and whether dosing and safety findings transfer across ages. It is not the right benchmark for every research question (Table 1, Section 4).

Figure 1 makes the gap visible directly, using Barkholtz and Bates’ own reported numbers rather than a schematic approximation. Panel A shows the age distribution of the general population reporting past-month cannabis use in 2021: a broad spread across four age bands from 18 to 64 (together 88.5 percent of past-month users, the remainder falling in age groups not shown), with no single age band dominating. Panel B shows the age distribution, at decade-level granularity, of participants enrolled in the cannabis pharmacokinetic and pharmacodynamic clinical trials that same analysis reviewed: a sharp concentration in the twenties, with just over half of age-documented participants falling in that single decade. The two panels are not built from identically sized age bins, because each reflects how its own source reported the data, so Panel C compares them on the coarsest cut points they share, and the comparison is best read as illustrative. A population spread broadly across adulthood is being studied through a participant pool concentrated in a single decade of it. In Panel C, adults aged 50 and older make up 12.3 percent of trial participants (ages 50 to 69; none older were reported) against at least 17.6 percent of past-month users, and 85.9 percent of trial participants are aged 18 to 49 against 70.9 percent of users. The difference on shared cut points is therefore more modest than the concentration in the twenties suggests. Panel B rests on 155 age-documented participants from six publications, mostly early-phase, healthy-volunteer designs that required prior cannabis exposure, so it illustrates enrollment in that trial class and does not represent cannabis research.

It is worth asking why a documented gap like this one persists three years on. Part of the answer is structural and not specific to cannabis. The Food and Drug Omnibus Reform Act of 2022 directed the FDA to require sponsors of covered drug trials to submit Diversity Action Plans specifying enrollment goals by age, sex, race, and ethnicity, a general mandate that would, if finalized and enforced, apply to future cannabinoid drug trials along with everything else [7]. As of this writing, that guidance remains unfinished: draft guidance was issued in 2024, removed from the FDA’s website in January 2025 amid a broader federal reconsideration of diversity-related agency guidance, restored by court order in February 2025, and has not yet been finalized past its original statutory deadline. Cannabis trials are not singled out by this uncertainty; they are simply not exempt from it. A field with a documented, quantified, three-year-old diversity gap cannot currently look to an external regulatory mandate to close it, at least not on any settled timeline, which makes the case for the field addressing the gap on its own terms, through the kind of reporting standard proposed in the final section of this piece, considerably stronger rather than weaker.

The next section makes this abstraction concrete. Clinical trial diversity gaps are easy to discuss as a statistical property of enrollment tables. They are harder to ignore once the population on the other side of the gap has names, diagnoses, and physicians they have not told.

## 6. Neurological Disease and Chronic Pain: The Population Cannabis Research Was Ostensibly for

If any population should anchor cannabis research, it is patients using cannabis to manage symptomatic neurological disease and chronic pain, since this is the use case most often invoked to justify legalization on medical rather than recreational grounds. It is also the population most consistently underrepresented in the evidence meant to guide its own care, which means the evidence base may have been built in large part from groups that differ from those using the drug therapeutically.

Multiple sclerosis illustrates the pattern cleanly. A 2026 systematic review and meta-analysis of cannabis-based therapies for MS-related spasticity found clinically meaningful improvement, particularly over longer treatment durations, but the authors themselves flagged high heterogeneity and suspected bias across the included trials as a reason for caution and called explicitly for further high-quality randomized trials with standardized protocols [8]. That caveat is heterogeneity suppression being named from inside the trial literature rather than diagnosed from outside it. Meanwhile, a multicenter cross-sectional survey of unprescribed cannabinoid use among Italian MS patients, conducted from March 2022 to February 2023, found a mean respondent age of 45.2 years and a sample that was 64.5 percent female [9], a demographic profile that does not obviously match the population enrolled in the spasticity trials the same review was synthesizing. The people using cannabis for MS symptoms and the people being studied to determine whether cannabis works for MS symptoms are not obviously the same people.

Parkinson’s disease shows the pattern in a more urgent form, because the mismatch here is not just demographic but behavioral. A Fox Insight survey of 1881 people with Parkinson’s, of which 58.5 percent were men and the mean age was 66.5, found that 73.0 percent reported medicinal cannabis use, yet 30.8 percent had not informed their physician [10]. Patients are not waiting for trial evidence to catch up. They are self-medicating, often successfully by their own report, with frequent improvements noted for pain, anxiety, agitation, and sleep, while keeping a third of that use invisible to the clinicians who would otherwise be positioned to screen for drug interactions or cognitive risk. A companion review on cannabinoids in late-life Parkinson’s disease and dementia is blunt about the evidence gap this creates: strong trial evidence remains scarce for most indications even as off-label use continues in routine clinical practice, and the review’s own recommendation, low starting doses with slow titration, is drawn from general geriatric prescribing principles rather than from cannabis-specific trial data in this population [11].

Epilepsy complicates the usual framing further, because it is often held up as cannabis research’s success story, the CBD-for-seizures narrative that predates most of the current legalization wave. But even here, heterogeneity suppression finds a foothold. A 2025 study examined the prevalence of cannabis use disorder specifically among privately insured adults with epilepsy [12], a population and a risk that receive almost no attention in a therapeutic narrative built around CBD’s low abuse potential. Treating cannabis and epilepsy as a single, settled, favorable story obscures the fact that people with epilepsy who use cannabis, including THC-containing products outside the CBD-specific formulations studied in seizure trials, are a distinct subgroup with a distinct risk profile that the dominant narrative does not track.

The throughline across all three conditions is not that cannabis fails these patients (several of the findings above suggest real symptomatic benefit) but that the evidence base has not organized itself around the population it claims to serve. Patients self-medicate, often without disclosing use to their physicians, and researchers subsequently build trials around whichever cohort is easiest to recruit rather than around the patients already using the drug. Self-report survey data of the kind cited throughout this section is doing real evidentiary work here not because it is methodologically preferable to a randomized trial, but because it is often the only data that reflects who is using cannabis for these conditions. That substitution, survey data standing in for trial data because trial data does not describe the relevant population, is itself a symptom of the mismatch this piece is describing. For these questions the relevant benchmark is the condition-specific population who use cannabis for that condition, not the general population of past-month users, and the surveys cited here serve that role. The scarcity of data in older adults is an evidence gap. It is not evidence of inefficacy or of harm.

## 7. Neuroimaging

Neuroimaging is the domain where the consequences of pooling by age are sharpest, but not for the reason one might expect. Unlike the psychiatric outcome literature discussed later in this piece, cannabis neuroimaging research has not entirely ignored older adults. Several well-powered studies now exist. The problem is that they disagree with each other in ways the field has not adequately reckoned with, and the disagreement may itself carry information about participant and exposure heterogeneity rather than being noise to be averaged away.

The UK Biobank has produced the most consistent picture, and it is not a favorable one. Vered, Sznitman, and Weinstein examined 19,932 dementia-free UK Biobank participants with a mean age of 68, and found cannabis use associated with smaller total, white matter, gray matter, and peripheral cortical gray matter volumes, with a clear dose–response pattern by duration and frequency of use, and with increased white matter hyperintensity volume among longer-duration users [13]. A related analysis by Ishrat and colleagues, combining observational and Mendelian randomization methods in a similarly aged UK Biobank cohort, reached a consistent conclusion while explicitly noting that older-adult cannabis users remain understudied relative to adolescents, an odd asymmetry given that this is the age band where use is growing fastest [14]. A follow-up paper from the same UK Biobank group extended the finding to cognitive function specifically, again in dementia-free adults 60 and older [15].

A University of Colorado Anschutz cohort, studying a wider and partly overlapping age range, found close to the opposite. Guha, Fu, Calhoun, and Hutchison examined lifetime cannabis use in adults aged 40 to 77 and reported larger gray matter volumes and faster processing speed among cannabis users relative to non-users [16]. A related preprint from an overlapping author group went further, describing overlapping brain network configurations between cannabis use and healthy aging, with effects the authors characterize as running in a reversed, favorable direction relative to typical age-related decline [17]. This preprint is not peer-reviewed and carries little weight in the argument that follows. These are not marginal findings dressed up as contradictions. They are large, methodologically serious studies, in overlapping but not identical age bands (UK Biobank: dementia-free, mean age of 68; Colorado: 40 to 77), pointing in opposite directions.

These findings can be read as an unresolved empirical question, and that remains a reasonable reading. A further possibility, offered here as a hypothesis, is that the studies differ in exposure and sampling in ways that matter. The cohorts differ in age range (UK Biobank: dementia-free adults, mean age of 68; Colorado: adults aged 40 to 77), in exposure metric (duration and frequency versus lifetime use), in recruitment (population invitation versus research volunteers), and plausibly in product potency and market context. They also differ in ways unrelated to heterogeneity suppression, including covariate adjustment, imaging acquisition and processing, and the possibility of residual confounding by alcohol, tobacco, education, and socioeconomic status. Both are cross-sectional, so reverse causation cannot be excluded, and exposure is self-reported. A comparison of two cohorts cannot determine which differences, if any, account for the divergence.

The Dual Diversity framework adds a testable prediction. If exposure and participant heterogeneity matter, then stratifying by product, potency, frequency, and age band within each cohort (Table 2), and re-analyzing both datasets with harmonized exposure definitions and covariates, should reduce or explain the discrepancy. If it does not, the explanation lies elsewhere. Systematic reviews that combine such cohorts should examine between-study heterogeneity and should not assume a single shared effect. A recent commentary on compounding validity threats in cannabis-aging neuroimaging develops this argument for the UK Biobank and related cohorts [18].

This matters beyond neuroimaging. The same mechanisms plausibly implicated in these structural findings (chronic low-grade neuroinflammation, blood–brain barrier integrity, and endocannabinoid tone) are the substance of the inflammation research taken up in the next section, and I have argued elsewhere that demographic composition needs to be treated as a validity threat rather than a statistical nuisance in exactly this kind of cannabinoid-inflammation work [4]. Disagreement in the direction of effect across older-adult cohorts suggests that the samples and exposures in these studies have not yet been characterized in enough detail to compare them directly. Whether market and cohort context contribute to the divergence is an open empirical question that harmonized, stratified analyses could address.

## 8. Inflammation and Immune Function

This is the section where my own prior argument applies most directly, since I have proposed elsewhere that demographic composition should be treated as a validity threat rather than a statistical nuisance specifically in cannabinoid-inflammation research [4]. The neuroimaging contradiction described in the previous section is not a self-contained puzzle. Structural brain volume, white matter integrity, and functional connectivity are downstream of processes (chronic low-grade neuroinflammation, blood–brain barrier permeability, and endocannabinoid system tone) that are themselves age-dependent in ways the inflammation literature has only begun to take seriously.

Aging changes the immune system in ways that are mechanistically relevant to cannabinoid action, not merely a source of added variance around it. A 2026 review of cannabinoid therapeutics in age-related pain notes that the inflammatory response in older adults is frequently dysregulated, shaped by immunosenescence and the low-grade chronic inflammatory state increasingly described in the aging literature, a baseline immune context that simply does not exist in the young, healthy volunteers who populate most cannabinoid-inflammation trial samples [19]. Testing an anti-inflammatory mechanism in a population whose inflammatory baseline is fundamentally different from the population the treatment is meant to serve is not a minor external validity concern. It may be closer to testing a drug in a different tissue context altogether.

The endocannabinoid system itself appears to change with age in ways that directly affect how cannabis use should be expected to act. A study assaying circulating endocannabinoids across three age bands, adults aged 21 to 24, 25 to 54, and 55 to 71, found that older adults exhibited lower baseline anandamide and other endocannabinoid levels than younger adults prior to any cannabis use, differences that were measurably ameliorated after acute cannabis use in the older group [20]. This is a rare example of a study that did the stratification this piece has been arguing for throughout, and the result is instructive precisely because it would have been invisible in a pooled sample. A study that averaged endocannabinoid tone across all three age bands would have reported a single number that described none of the three groups particularly well and would have missed the finding that gives the paper its clinical relevance: that cannabis use may be doing something age-specific, restoring a baseline deficit in older users rather than producing a uniform effect across the lifespan.

Mechanistic reviews of cannabinoid dosing in aging-related neurobiological change describe a similar picture from the other direction, cataloging how neuroinflammation, reduced neurogenesis, impaired neuroplasticity, and blood–brain barrier dysfunction accumulate with age and interact with cannabinoid signaling in ways that are still poorly characterized in humans [21]. At least one recent study has begun testing combination approaches built explicitly around this age-related complexity: a trial pairing THC with celecoxib to capture THC’s anti-inflammatory and neuroprotective potential while offsetting its known cognitive costs, a design that only makes sense once one accepts that an older, more cognitively vulnerable population requires a different risk–benefit calculation than the young recreational users in whom most THC pharmacology was originally characterized [22].

That trial is early and narrow, but it represents the kind of age-conscious design this piece is arguing the field needs considerably more of, not as an exception, but as a default.

Taken together, these findings point toward the same conclusion the neuroimaging section reached from a different direction. Age is not a covariate to be adjusted for in cannabinoid-inflammation research. It changes the immune and endocannabinoid context the drug is acting on, which means a study population’s age composition is part of what determines which biological question is being asked. A field that continues to draw inflammation-focused cannabinoid samples primarily from young, healthy volunteers is not producing findings that generalize poorly to older adults. It is producing findings that may not describe the biology of aging cannabis users at all.

## 9. Psychiatry and Psychosis Risk

The heterogeneity suppression problem in the neuroimaging and inflammation sections operates mostly within studies, pooled samples averaging away subgroup differences that were present in the data all along. The psychosis-risk literature exhibits a different and, in some ways, more stubborn version of the same problem: heterogeneity suppression at the level of an entire subfield’s sampling frame. Ask what age band cannabis psychosis research is conducted in, and the answer across nearly every 2025 and 2026 study identified for this review is the same: adolescence and young adulthood, almost without exception.

The scale of that concentration is worth stating plainly. A 2026 cohort study drew on 463,396 adolescents aged 13 to 17, universally screened for cannabis use, and found past-year use associated with significantly increased risk of clinician-diagnosed psychotic, bipolar, depressive, and anxiety disorders through age 26, with the strongest associations for psychotic and bipolar disorders, robust after adjustment for sociodemographics, other substance use, and prior psychiatric history [23]. This is a genuinely well-powered, well-designed study, and its findings are not in question here. What is worth noting is what an evidence base built almost entirely from studies like this one cannot tell a clinician anything about psychosis risk in a 68-year-old initiating cannabis use for the first time, or resuming it after a decades-long gap, using a modern high-potency product that bears little resemblance to what may have been available in that person’s own adolescence.

That last point matters because the field’s own explanation for rising psychosis risk is itself a demographic and historical argument, not a fixed pharmacological one. A 2025 review in a major medical journal ties the increasing link between cannabis use and psychosis and schizophrenia directly to product potency, noting that THC content in commonly available cannabis has increased roughly fivefold over the past two decades in some legal markets [24]. If the potency era is doing real explanatory work, and the reviewers who study this most closely argue that it is, then a person initiating cannabis use in their sixties on a 2026-era high-potency product may not be replicating the risk profile established by adolescent-onset studies from the 2000s and 2010s, nor is that person captured by the adolescent cohorts driving current psychosis-risk estimates. Direct evidence on that population appears to be lacking, and no inference about risk in either direction is supported by the current literature. A systematic review and meta-analysis of psychosis risk thresholds has already established that age of onset, potency, and frequency of use function as compounding, not merely additive, risk factors, with early-onset frequent high-potency use carrying substantially higher risk than any one factor alone [25]. The field, in other words, already knows heterogeneity by age and product era matters enormously to this outcome. It simply has almost no data on how those known compounding factors behave in a population initiating use for the first time in late life, on today’s products, a population Section 3 of this piece shows is not hypothetical but rapidly growing. The potency argument is a rationale for studying late-life initiators. It is not evidence that risk in that group is higher, or lower.

Even the literature’s methodologically sophisticated corners show the same concentration. A systematic review examining dissociative experience and self-disturbance in first-episode psychosis, cannabis use, and their interaction drew on 22 studies and nearly 3850 participants, a genuinely substantial evidence base by the standards of this subfield [26]. It is, by its own inclusion criteria, entirely a first episode and therefore predominantly young-adult literature. That is not a flaw in the review. It is an accurate reflection of where the underlying primary research has been conducted, which is precisely the point. A field can build increasingly sophisticated, well-powered, methodologically careful reviews indefinitely within a single age band without ever producing evidence relevant to psychosis risk in the population now most rapidly taking up cannabis use.

I have argued in other contexts that symptom equivalence does not imply neurobiological equivalence, that two people meeting the same diagnostic or exposure criteria may be instantiating that category through meaningfully different underlying biology. The same logic applies here with the terms reversed: exposure equivalence does not imply risk equivalence. Cannabis use in a 17-year-old on a high-potency product with a family history of psychotic illness and cannabis use in a 70-year-old initiating low-frequency use of a CBD-dominant product for chronic pain are not the same exposure (Table 2 lists the exposure dimensions that distinguish them) and treating psychosis-risk findings from the first population as informative, even qualitatively, about the second is an inference that current evidence does not support. The next section turns to what happens in ordinary clinical practice when this gap meets a health system that is, for entirely separate reasons, not routinely asking older patients about cannabis use in the first place.

## 10. Aging, Polypharmacy, and the Screening Gap

The preceding sections have described an evidence base that has not kept pace with an aging cannabis-using population. This section describes what that gap looks like at the point of care, where the consequences stop being academic. An older adult using cannabis is, by the numbers in Section 3, increasingly common, and that same older adult is disproportionately likely to be doing so alongside other medications, with a physician who has not asked and may not know.

The clinical stakes are not hypothetical. Among veterans aged 65 to 84 in a cross-sectional VHA sample, 10 percent reported past-30-day cannabis use, and among those users, 36 percent met criteria for cannabis use disorder [2]. That finding required researchers to specifically ask a structured, validated question set in a population sampled for that purpose. Most primary care encounters do not include an equivalent step. General adult substance use screening is a recognized standard, the US Preventive Services Task Force recommends screening when accurate diagnosis and follow-up treatment can be offered, and a cluster-randomized implementation trial has tested population-based screening and symptom assessment for cannabis and other drug use across primary care sites [27]. But general-adult screening protocols were not designed around, and do not specifically flag, the particular risk profile of an older patient combining cannabis with a geriatric medication regimen, which is a different clinical question than identifying cannabis use disorder in a younger population.

A small number of programs are beginning to build the more targeted alternative. A geriatric primary care clinic recently described embedding a physician-led medical cannabis clinic directly within its practice, with structured evaluation that includes reviewing all documented medications against at least two independent drug interaction databases before the clinical encounter [28]. The preliminary data from that model are themselves informative: patients served had high clinical complexity and frequent identified drug interactions, precisely the pattern one would predict given the polypharmacy burden common in geriatric populations, and precisely the pattern that goes undetected in a standard visit that does not ask about cannabis at all. The existence of this model is encouraging. Its rarity is the more important finding. A structured, interaction-aware approach to medical cannabis in older adults is currently a novel pilot program worth publishing, not a baseline standard of care.

The pharmacological logic for concern is not speculative. Cannabinoids are metabolized substantially through the same hepatic cytochrome P450 pathways, particularly CYP3A4 and CYP2C9, that govern the metabolism of warfarin, many benzodiazepines, and a range of other medications disproportionately prescribed to older adults. Aging itself alters hepatic and renal clearance in ways that can shift the therapeutic margin for interacting drugs even before cannabis is added to the regimen. None of this requires a novel finding to state; it follows from established geriatric pharmacology once the premise, that a meaningful fraction of older patients is using cannabis, is taken seriously as a starting assumption for a clinical encounter rather than an incidental detail a patient might or might not volunteer.

Candidate screening instruments exist but are not tailored to older adults. The WHO ASSIST and its brief adaptations, the Cannabis Use Disorders Identification Test—Revised (CUDIT-R), and single-question screens could be adapted for geriatric and primary care settings, although validation in adults aged 65 and older is limited, and age-specific validation is a research priority. At the system level, electronic health records could carry structured fields for cannabis use (product type, THC:CBD ratio, route, and frequency) linked to interaction alerts for CYP3A4 and CYP2C9 substrates such as warfarin. These are proposals, not established practice. The clinical value of such alerts, their burden on clinicians, and the accuracy of patient-reported product information all need evaluation before implementation.

This is where the abstractions of the preceding sections resolve into something a treating physician must act on regardless of whether the research base has caught up. A neuroimaging literature that cannot agree on a direction of effect, a psychosis-risk literature built on adolescent cohorts, and an inflammation literature drawn from young healthy volunteers all fail, in their different ways, to tell a clinician what to expect when an older patient on four medications reports starting cannabis for sleep or pain. The screening gap does not wait for that literature to resolve itself. This is where the diversity gaps with which this piece opened stop being a property of enrollment tables and become a property of what happens, or does not happen, in an exam room. Section 11 turns to what a research base that had kept pace would need to do differently to close that gap rather than simply describing it again.

## 11. What Non-Dazed Research Would Look Like

Every section of this piece has described a version of the same failure: a research base whose sample composition has not moved with the population it studies, in ways that are not marginal to the findings but constitutive of them. It is worth being concrete about what closing that gap would require, because the alternative to the current approach is not an impossible standard. Much of it already exists as a template elsewhere in biomedical research and simply has not been applied here.

The first requirement is demographic composition reporting benchmarked against population use, not just sample description in isolation. Barkholtz and Bates already demonstrated the method: compare trial or study demographics directly against national survey data on who uses cannabis, rather than reporting sample demographics as a table without a population referent [3]. This should be a standard reporting element for cannabis research, not an occasional methods paper. A sample that is 90 percent under age 40 means something different, and something reportable, when the population using the substance under study is distributed broadly across adult life.

The second is age-stratified minimum enrollment, and here the field does not need to invent a framework from scratch. The ICH E7 guideline on studies in support of geriatric populations has required special consideration of pharmacokinetic, pharmacodynamic, and drug–drug interaction differences in elderly patients for pharmaceutical development since 1993, with FDA guidance reaffirming and updating those expectations as recently as 2012 [29]. That guideline exists precisely because regulators recognized decades ago that a drug’s effects in a geriatric population cannot be safely extrapolated from a younger trial cohort. Cannabis research has largely operated outside this expectation, not because the underlying pharmacological logic does not apply (it plainly does) but because most cannabis products used by patients sit outside the FDA drug approval pathway that would trigger ICH E7-style requirements in the first place. A geriatric-specific study requirement already exists as a matter of established regulatory science. Cannabis research’s exemption from it appears to follow from the substance’s unusual regulatory status and not from a considered judgment that the pharmacology does not warrant it.

The third is heterogeneity-preserving analysis: reporting subgroup effects rather than collapsing a diverse population into a single cannabis user main effect. The endocannabinoid tone study discussed in the inflammation section demonstrates exactly what this looks like in practice, and exactly what would have been lost without it: a finding that was only visible because the researchers stratified by age band rather than reporting a single pooled result [20]. That should be the default analytic posture for a heterogeneous exposure like cannabis, not a special design choice that happens to get published when it produces an interesting result.

The fourth is a role for journals themselves. A journal that already asks authors to justify demographic composition and address it as a potential validity threat, rather than treating it as an optional footnote, changes incentives at the point where they are most likely to matter, before a study is designed rather than after it is published and its limitations are noted in a single sentence [4]. This does not require new infrastructure. It requires treating demographic reporting with the same rigor already applied to statistical methods and conflicts of interest disclosure.

The fifth is a minimum exposure descriptor set: reporting product composition (THC and CBD content), potency, route, dose and frequency, duration and cumulative exposure, age at initiation, purpose of use, and whether the supply was regulated and tested (Table 2). Without these descriptors, studies cannot be stratified or compared, and exposure misclassification is likely to distort results in unpredictable ways.

None of these five recommendations is difficult to state, and none of them is original to cannabis research specifically. What is specific to cannabis research is the speed and scale of the population shift these recommendations would need to catch up to, and the fact that the population currently least represented in the evidence, older adults managing neurological disease, chronic pain, and the ordinary comorbidities of aging, is precisely the population with the least room for error.

Table 3 maps each recommendation to its methodological objective, primary audience, and relevant guideline (ICH E7 [29], CONSORT [30], STROBE [31]).

## 12. Limitations

This review has several limitations. First, it is a narrative review without preregistration, formal risk-of-bias appraisal, or pooled estimates, and source selection was purposive. Although contrary findings were sought, selection may favor studies consistent with the argument. Second, the epidemiological and regulatory material is predominantly from the United States (NSDUH, FDA, FDORA). Generalization to Europe and Canada, where legal frameworks, product markets, and screening practices differ, is uncertain. Third, the quantitative anchor is a single secondary analysis with data through 2021, and the trial age distribution rests on 155 participants from six publications. Fourth, several cited findings are cross-sectional or self-reported, and one is a preprint.

Fifth, the review does not evaluate the barriers to enrolling older, multimorbid participants, which may explain part of the diversity gap. These include ethical barriers (consent and capacity), logistical barriers (transport, caregiver support), clinical barriers (exclusion for polypharmacy, frailty, and comorbidity), and economic barriers (cost of monitoring and longer follow-up). The recommendations, particularly age-stratified enrollment, carry costs that are not assessed here and have not been tested. Finally, the scarcity of data in older adults is an evidence gap. It is not evidence of inefficacy or of harm.

## 13. Closing

The title of this piece is a joke with a methodological point underneath it. Cannabis research is not dazed and confused in the sense the phrase originally means, disoriented, uncertain, and groping toward an answer it cannot yet see. The evidence base described across these five domains is often careful, well-funded, and methodologically serious on its own terms. That is what makes the mismatch worth naming rather than dismissing. A field can be confident, well-resourced, and rigorous by every internal standard it holds itself to and still describe a population that differs from the one now using cannabis, because the mismatch lives in the sampling frame rather than in any individual study’s statistics.

The corrective this piece has argued for is not a call to distrust cannabis research, nor a case against its therapeutic promise; several of the findings surveyed here suggest real benefit for real patients. It is a case for taking the population using cannabis today, which is older, more medically complex, and more often managing a diagnosed neurological condition or chronic pain than the field’s founding samples ever anticipated, as seriously as the substance itself has already been taken. The demographic drift documented in Section 3 is not going to reverse. The research base that describes it can still choose to catch up.

## Figures and Tables

**Table 2:** Dimensions of cannabis exposure and minimum reporting.

Dimension	Why It Changes the Exposure	Minimum Descriptor to Report
THC and CBD composition	THC and CBD have different and partly opposing pharmacology	THC and CBD content per product (mg or percent)
Potency	THC concentration differs by market and era	Product THC content and study period
Route of administration	Inhaled and oral routes differ in onset, peak, and duration	Route or routes, and proportion of use by route
Dose and frequency	Effects and risks scale with both	Typical dose per session and days of use per month
Duration and cumulative exposure	Brain and cognitive findings track duration	Years of use and a cumulative exposure estimate
Age at initiation	Developmental timing and late-life initiation carry different risk	Age at first use, and any gaps in use
Purpose of use	Medicinal and recreational users differ in dose, product, and comorbidity	Primary purpose and target condition
Regulated versus unregulated supply	Content is verified in one and uncertain in the other	Source of product and whether it was laboratory tested

**Table 3:** Recommendations, objectives, audiences, and guideline anchors.

Recommendation	Methodological Objective	Primary Audience	Guideline Anchor
1. Report sample demographics against population surveillance data	Quantify selection and transportability	Researchers, journals	CONSORT item 15; STROBE item 14; FDORA Diversity Action Plans (US)
2. Age-stratified minimum enrollment	Adequate precision in older strata; estimation of effect modification	Researchers, ethics committees, sponsors	ICH E7
3. Heterogeneity-preserving analysis	Report subgroup and interaction estimates instead of one pooled effect	Researchers, statisticians	CONSORT item 18; STROBE item 12b
4. Justification of demographic composition at submission and ethics review	Change incentives before study design	Journals, ethics committees	Journal policy; CONSORT and STROBE reporting items
5. Minimum exposure descriptor set	Reduce exposure misclassification and enable stratification	Researchers, journals	STROBE item 7; Table 2

## Data Availability

No data was used for the research described in the article.

## Supplementary Materials

The following supporting information can be downloaded at https://www.mdpi.com/article/10.3390/life16101712/s1. File S1: Full PubMed search strings for the combined cannabis and age search and for each review domain.
