# Supplementary material for: Still Dazed and Confused: Demographic Drift and the Persistence of Sampling Bias in Cannabis Research
Source: Life (Basel). 2026 Oct 13;16(10):1712. doi: 10.3390/life16101712 (PMC13706348; doi:10.3390/life16101712)
Supplement: Supplementary file 1 [file life-16-01712-s001.zip › life-4611782-supplementary.pdf]

Supplement: PubMed search strings

Database: PubMed. Date window: 1 January 2020 to 6 October 2026, applied with the publication date field ([dp]). Run each domain string as: Cannabis block AND Age block AND Domain block AND Date filter (no quotation marks around the date range). Hit counts in Table S1 are the number of records in the CSV exports downloaded on 6 October 2026.

Cannabis block

(cannabis[tiab] OR cannabinoid*[tiab] OR marijuana[tiab] OR cannabidiol[tiab] OR THC[tiab])

Age block

("older adult*"[tiab] OR elderly[tiab] OR aging[tiab] OR ageing[tiab] OR geriatric*[tiab])

Date filter

2020/01/01:2026/10/06[dp]

Domain	Domain block	Hits	Excluded	Unclear	Carried forward
Cannabis and age (all domains)	none (Cannabis block AND Age block AND Date filter (no quotation marks around the date range))	763	102	205	456
Trial diversity and sampling	(representativ*[tiab] OR diversity[tiab] OR demographic*[tiab] OR "sampling bias"[tiab]) AND trial*[tiab]	4	0	0	4
Neurological disease and chronic pain	("multiple sclerosis"[tiab] OR parkinson*[tiab] OR epilepsy[tiab] OR "chronic pain"[tiab])	75	12	13	50
Neuroimaging	(neuroimaging[tiab] OR MRI[tiab] OR "brain structure"[tiab] OR "gray matter"[tiab] OR "grey matter"[tiab] OR "UK Biobank"[tiab])	20	0	4	16
Inflammation and immune function	(inflammat*[tiab] OR immun*[tiab] OR endocannabinoid*[tiab])	204	55	46	103
Psychosis risk	(psychosis[tiab] OR psychotic[tiab] OR schizophrenia[tiab])	13	4	6	3
Polypharmacy	(polypharmacy[tiab] OR "drug	74	4	32	38

Domain	Domain block	Hits	Exclude d	Uncle ar	Carried forward
and screening	interaction*"[tiab] OR screening[tiab])				

Additional identification. Forward and backward citation searching from Barkholtz and Bates (2023) and from the primary studies in each domain. Older foundational sources (ICH E7, NSDUH trend data from 2002) were identified through citation searching and regulatory sources and fall outside the dated search window.

Eligibility. Peer-reviewed human studies, systematic reviews, and regulatory documents in English reporting participant age composition or age-related outcomes. Excluded: preclinical-only studies, case reports, and studies without age information. Preprints included only when labeled and not used for a central claim.

Screening notes (Table S1). Domain searches are subsets of the combined cannabis and age search, so the 763 unique records are not the sum of the domain rows. Screening was at title level with AI assistance followed by author review. Excluded: preclinical or non-human (72), adolescent or young-adult only (23), case reports or series (7). Unclear: no cannabis term in the title, not systematically assessed. Carried forward: candidate records for narrative selection, not all of which were cited. Of the 26 journal articles cited in the manuscript other than reporting guidelines, 14 were retrieved by these searches. The full record-level decisions are in Screening_results.csv.
